# MicroRNA-1258: An invasion and metastasis regulator that targets heparanase in gastric cancer

**DOI:** 10.3892/ol.2021.13103

**Published:** 2021-10-20

**Authors:** Jinxin Shi, Ping Chen, Jingxu Sun, Yongxi Song, Bin Ma, Peng Gao, Xiaowan Chen, Zhenning Wang

Oncol Lett 13: 3739-3745, 2017; DOI: 10.3892/ol.2017.5886

Subsequently to the publication of the above article, the authors have realized that the labels for Figure parts 1B and 1C were printed the wrong way around; furthermore, a pair of the data panels were inadvertently featured in Fig. 2 containing overlapping data which were derived from the same original source, even though the data were intended to have represented different experimental conditions.

A correctly labelled version of Fig. 1 is shown below, and the corrected version of Fig. 2 is shown on the next page, featuring the correct data for the SGC-7901 experiment. Note that the errors in the Figure did not affect either the results or the conclusions reported in this study. The authors are grateful to the Editor of *Oncology Letters* for granting them the opportunity to publish this corrigendum, and regret any inconvenience caused to the readership of the Journal.

## Figures and Tables

**Figure 1.**
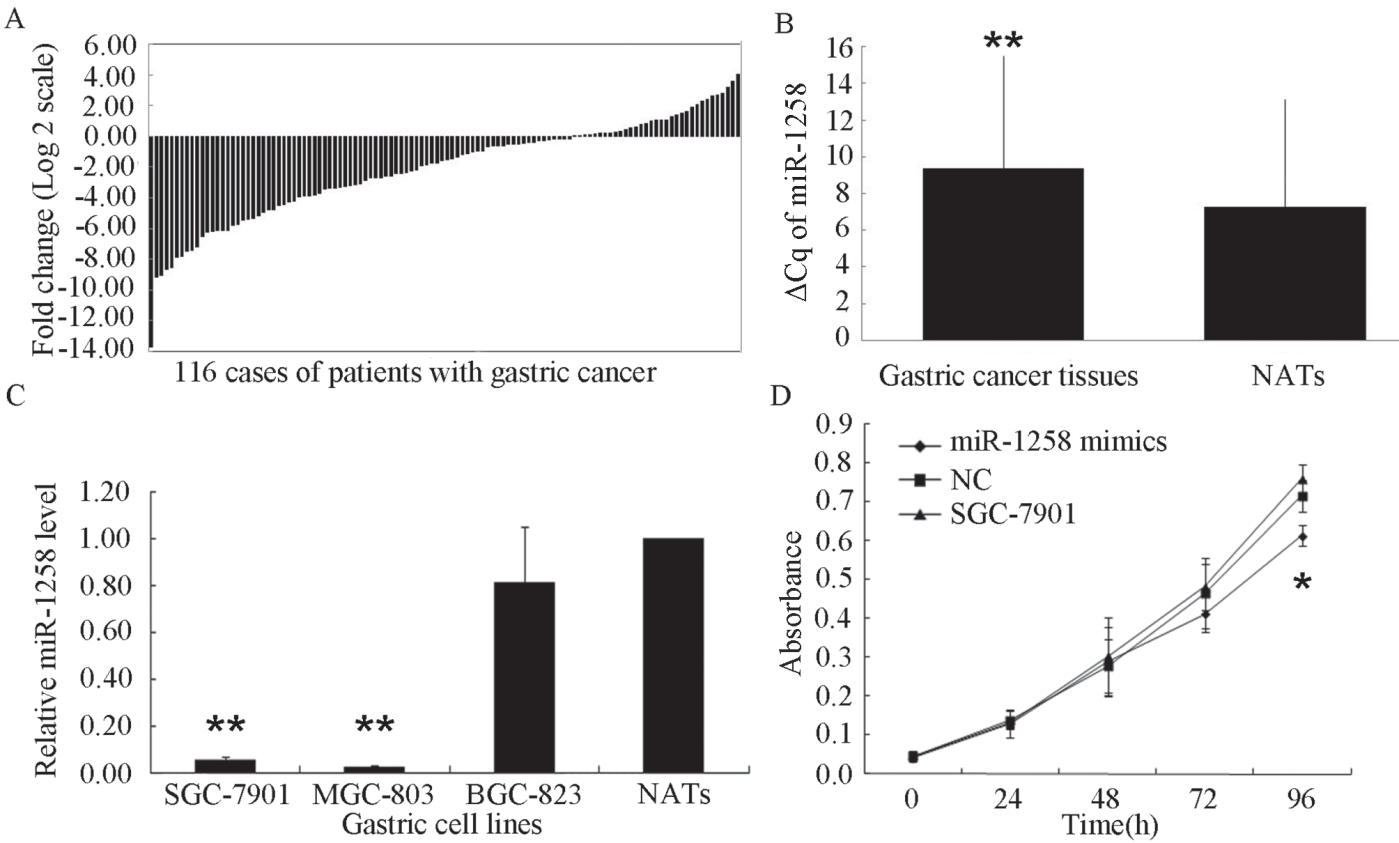
Expression of miR-1258 in tissues and cell lines. (A) miR-1258 was detected in 116 patients with gastric cancer using the reverse transcription-quantitative polymerase chain reaction. Results are presented as log2 fold change of gastric cancer relative to NATs. (B) Significantly increased ΔCq values for miR-1258 were observed between gastric cancer tissues and NATs. (C) Mean expression of miR-1258 in gastric cancer cell lines (MGC-803, SGC-7901 and BGC-823) relative to NATs. (D) MTT proliferation assay in SGC-7901 cells. *P<0.05; **P<0.01 vs. NAT. miR-1258, microRNA-1258; NAT, non-tumor adjacent tissue; NC, negative control.

**Figure 2.**
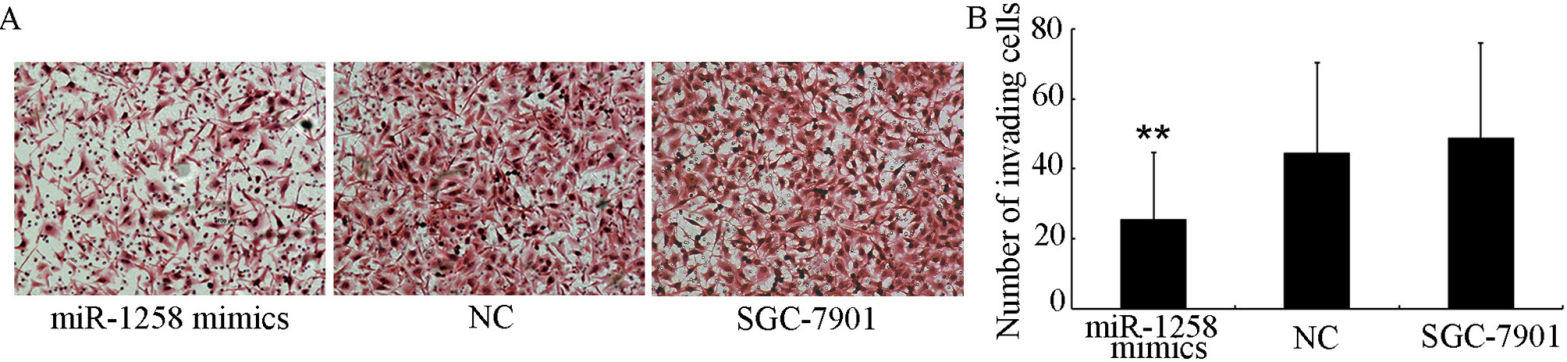
Overexpression of miR-1258 suppresses SGC-7901 cell invasion *in vitro*. A Transwell invasion assay was performed to estimate the effect of miR-1258 on the invasive potential of gastric cancer cells. (A) Representative images and (B) quantification of the cells that had migrated to the basal side of the membrane. Magnification, ×200. **P<0.01, miR-1258 transfected vs. untransfected SGC-7901 cells. miR-1258, microRNA-1258; NC, negative control.

